# Effects of liraglutide on blood pressure and renal function in patients with type 2 diabetic nephropathy: a meta-analysis of randomized controlled trials

**DOI:** 10.3389/fmed.2026.1921737

**Published:** 2026-10-09

**Authors:** Shengning Gao, Yudong Shan, Dongxue Ma, Xiaohong Zhou, Weijuan Gao

**Affiliations:** 1 Hebei University of Chinese Medicine, Shijiazhuang, Hebei, China; 2 Hebei Key Laboratory of Chinese Medicine Research on Cardio-Cerebrovascular Disease, Hebei University of Chinese Medicine, Shijiazhuang, Hebei, China

**Keywords:** blood pressure, liraglutide, meta-analysis, systematic review, type 2 diabetic nephropathy

## Abstract

**Objectives:**

The effects of liraglutide on blood pressure in patients with T2DN remain unclear. This meta-analysis aimed to systematically evaluate the effects of liraglutide on blood pressure and renal function in patients with T2DN.

**Methods:**

We conducted comprehensive searches of Web of Science, PubMed, the Cochrane Library, Elsevier ScienceDirect, CNKI, Wanfang Data, and VIP databases. Eligible studies were RCTs of ≥12 weeks’ duration evaluating liraglutide for T2DN. Two independent reviewers screened citations, extracted data, and assessed methodological quality using the Cochrane Risk-of-Bias Tool. Statistical analyses were performed in Stata 17.0 and RevMan 5.4, with effect sizes presented as RR or MD and 95% CI. The Cochrane Q test and I^2^ statistic were used to quantify statistical heterogeneity.

**Results:**

This meta-analysis included 23 RCTs involving 1,723 participants. Compared with the control group, liraglutide significantly reduced SBP [MD = −5.00, 95% CI (−7.17, −2.84)], while no significant effect was observed on DBP. In addition, liraglutide was associated with favorable changes in some renal biomarkers, as evidenced by reductions in Scr, UACR, Cys-C, BUN, and *β*2-MG, as well as elevations in eGFR. Improvements were also observed in glycemic control, lipid profiles, BMI, and inflammatory markers. Subgroup analyses indicated dose-dependent differences in UACR and LDL-C. In addition, both UACR and Scr were affected by intervention-regimen type, while HbA1c was affected by baseline albuminuria category. Sensitivity analyses confirmed the robustness of the findings, and adjustment for publication bias did not alter the statistical significance of the key outcomes.

**Conclusion:**

Liraglutide may be associated with favorable changes in selected BP, renal, metabolic, and inflammatory outcomes in patients with T2DN. However, further high-quality studies are needed to confirm these findings.

**Systematic Review Registration:**

https://www.crd.york.ac.uk/PROSPERO/view/CRD420251232844, PROSPERO CRD420251232844.

## 1 Background

Type 2 diabetic nephropathy (T2DN) is a major microvascular complication of type 2 diabetes mellitus (T2DM) (1, 2). Nearly 40% of T2DM patients globally now develop DN. The recent transformation in global lifestyles has led to a significant increase in both the incidence and mortality of diabetes, putting a greater strain on national economies (3, 4). DN, a major cause of end-stage renal disease (ESRD), initially presents as elevated urinary protein excretion and eventually progresses to renal failure due to a steady decline in glomerular filtration rate (GFR) (5–7).

The pathogenesis of DN typically involves multiple factors, including inflammation, hemodynamic abnormalities, and impaired lipid and carbohydrate metabolism (8). Chronic disturbances in glycemic and lipid balance promote the production of reactive oxygen species, resulting in oxidative stress and inflammation. This process gradually exacerbates renal immune cell infiltration, cytokine secretion, and renal fibrosis progression. Oxidative stress and inflammation act synergistically to accelerate renal injury and promote disease progression (9–11). Moreover, dyslipidemia may contribute to the development or progression of DN (12). Lipid metabolism issues can harm endothelial cells and podocytes, hastening the progression of glomerular sclerosis (13, 14). Current evidence suggests that cholesterol-associated markers may predict the development of DN (15). The risk of DN can be successfully reduced by increasing control over lipid profiles (16).

Hypertension is not only a common comorbidity of DN but also a key contributor to its onset and progression. Renal damage brought on by diabetes directly causes high blood pressure (BP), which worsens the hemodynamic strain on the renal system and accelerates renal function decline. As a result, in clinical practice, hypertension is a crucial target that needs focused attention. Raised BP in DN cannot be attributed to a single factor; increased activity of the renin-angiotensin-aldosterone system (RAAS), the sympathetic nervous system, and elevated levels of asymmetric dimethylarginine are all significant drivers of the development of hypertension in DN (17). Research has shown that in certain DN patients, rigorous antihypertensive treatment can lower albuminuria and decrease the rate of GFR deterioration (18). Currently, first-line therapy for DN is primarily based on angiotensin receptor blockers (ARBs) or angiotensin-converting enzyme inhibitors (ACEIs). These agents slow the progression of renal disease mainly by reducing intraglomerular pressure through inhibition of the RAAS. Nevertheless, BP control in some patients remains inadequate and fails to delay the progression of the disease. Glucagon-like peptide-1 receptor agonists (GLP-1RAs) have been increasingly used to manage diabetes and its complications in recent years, with liraglutide being widely applied in the treatment of DN. Beyond its glucose-lowering effect, liraglutide has been reported to exert multiple benefits, including weight reduction, anti-inflammatory effects, and renal protection (19, 20). However, whether liraglutide influences BP in patients with DN has not yet been comprehensively evaluated.

A preliminary literature search revealed that two meta-analyses were published to evaluate the therapeutic effect of liraglutide for T2DN (21, 22). Both meta-analyses focused on the therapeutic efficacy of liraglutide, but notable limitations remain. Firstly, the impact of liraglutide on BP has not been sufficiently evaluated. To comprehensively assess the therapeutic effects of liraglutide in T2DN, it is essential to include BP as a treatment endpoint. While liraglutide's cardiovascular benefits are well established, its exact role in BP regulation among patients with T2DN remains unclear (23, 24). Secondly, previous meta-analyses included trials with relatively short treatment durations, with some intervention periods as short as 4–8 weeks. As DN is a chronic progressive disorder, pharmacological agents rarely exert full therapeutic effects in the short term. Thus, assessing the short- to medium-term effects of drugs is crucial for DN management. Inclusion of short-duration trials of 4–8 weeks may increase heterogeneity. Presently, systematic evaluations specifically addressing the short- to medium-term effects of drugs are lacking. Third, the evaluation of certain clinically relevant biomarkers remains incomplete. For example, inflammatory markers such as high-sensitivity C-reactive protein (hs-CRP) and lipid parameters including low-density lipoprotein cholesterol (LDL-C) were not assessed, but both play important roles in the development and progression of DN. Elevated hs-CRP and LDL-C have been associated with DN severity and may reflect disease progression. Therefore, whether liraglutide improves BP and renal biomarkers in T2DN remains controversial.

Recently, there has been increasing interest in the effects of liraglutide on blood pressure in T2DN, and BP has gradually been incorporated as an outcome measure in clinical studies. Therefore, the present study included randomized controlled trials (RCTs) published from database inception to September 2026 to systematically evaluate the effects of liraglutide on BP and related indicators in patients with T2DN. This meta-analysis exclusively included RCTs with intervention durations of at least 12 weeks. Using this approach, we aimed to clarify the therapeutic benefits of liraglutide and overcome the limitations of short-term studies, which may not adequately capture changes in BP and renal function markers. Notably, this study represents the first attempt to assess the impact of liraglutide on BP parameters in T2DN. In addition, it overcomes the limitations of previous studies related to short follow-up durations and incomplete assessment of outcome indicators, thereby providing important evidence for a more comprehensive evaluation of the short- to medium-term therapeutic effects of liraglutide on glycemic control, BP, lipid metabolism, inflammation, and renal function.

## 2 Methods

### 2.1 Protocol registration and reporting

Registration of this meta-analysis was completed via PROSPERO (International Prospective Register of Systematic Reviews), with the assigned registration number CRD420251232844. We maintained adherence to the PRISMA statement and its outcome reporting extension guidelines throughout the study (25).

### 2.2 Search strategy

Electronic database searches in PubMed, Web of Science, Cochrane Library, Elsevier ScienceDirect, CNKI, Wanfang Data, and VIP Database were used to find papers. All fields used the following Medical Subject Headings and/or key terms: “liraglutide,” “diabetic nephropathy,” “Diabetic Kidney Disease,” “Diabetic Kidney Diseases,” “Diabetic Glomerulosclerosis,” “Intracapillary Glomerulosclerosis,” “Kimmelstiel Wilson Disease,” “Nodular Glomerulosclerosis,” and “Kimmelstiel Wilson Syndrome.” All databases were searched from inception to September 7, 2026. Supplementary Table 1 provides a detailed search strategy.

Additionally, the reference lists of relevant studies and systematic reviews were manually screened to retrieve potential eligible studies not captured by electronic database searches. Two independent reviewers (Shan YD, Ma DX) performed title/abstract screening and full-text eligibility assessment independently. Disagreements were resolved through consensus discussion.

### 2.3 Inclusion and exclusion criteria

Inclusion Criteria

- Study design: RCTs;
- Participants: adults (≥18 years old) with clinically confirmed T2DN. The diagnosis of T2DN was based on the criteria reported in the original trials. All included participants had T2DM and evidence of diabetic kidney involvement, including increased urinary albumin excretion [urinary albumin-to-creatinine ratio (UACR) ≥ 30 mg/g], proteinuria, or a reduced eGFR.
- Study treatment duration: at least 12 weeks;
- Intervention: The experimental group comprises patients treated with liraglutide, while the control group receives conventional antihypertensive or other hypoglycemic agents with no exposure to liraglutide;
- Studies were required to report at least one prespecified outcome, including glycated hemoglobin A1c (HbA1c), body mass index (BMI), UACR, eGFR, blood urea nitrogen (BUN), serum creatinine (Scr), total cholesterol (TC), triglycerides (TG), LDL-C, systolic blood pressure (SBP), diastolic blood pressure (DBP), hs-CRP, Cystatin C (Cys-C), *β*2-microglobulin (*β*2-MG).

Exclusion Criteria

- Non-RCTs, retrospective research, animal/cell experiments, and systematic articles;
- Study participants not explicitly diagnosed with T2DN;
- Trial intervention duration of < 12 weeks;
- Studies with defective data, incomplete outcome indicators, or failure to acquire supplementary data from the corresponding authors;
- Duplicate publications.

### 2.4 Data extraction

Data extraction was conducted for studies meeting the inclusion criteria by two independent researchers (Shan YD, Ma DX). Any disagreements were resolved by consensus among all authors.

Extracted data included:

- title, first author, and publication year;
- study design and treatment duration;
- sample size, mean age, gender ratio, and disease duration;
- administration details for study medications;
- primary outcome indicators (SBP, DBP, UACR); secondary outcome indicators (HbA1c, TC, TG, LDL-C, BMI, hs-CRP, Scr, eGFR, BUN, Cys-C, *β*2-MG)

For studies with unclear published data, the corresponding authors were contacted to obtain additional information where necessary; disagreements during data extraction were resolved by consensus.

### 2.5 Risk of bias assessment

We organized our literature references using Zotero 7.0.27. Using the Cochrane Risk-of-Bias Tool, two authors, Shan YD and Ma DX, independently assessed the methodological reliability of RCTs, working in duplicate to cross-validate their assessments. Seven domains were covered in the assessment aspect: random sequence generation, allocation concealment, blinding of participants and personnel, blinding of outcome assessment, completeness of outcome data, selective reporting, and other biases. Each study's methodological quality was divided into “high risk”, “unclear risk”, and “low risk” levels.

### 2.6 Statistical analyses

When the standard deviation of the mean change was not reported in the included RCTs, Shan YD and Ma DX calculated the change values for the relevant outcomes and incorporated them into the meta-analysis. The corresponding formula is shown in Supplementary Table 2. When multiple intervention groups were reported, only the group consistent with the inclusion criteria was included in the analysis. We performed statistical analyses using RevMan 5.4 and Stata 17.0. Forest and funnel plots were constructed with RevMan 5.4, whereas sensitivity analyses and Egger's test were conducted using Stata 17.0. Subgroup analyses were conducted to explore potential sources of heterogeneity. Effect measurements for dichotomous variables were represented as relative risks (RR) or odds ratios (OR), with matching 95% confidence intervals (CI) computed for each. For continuous variables, mean differences (MD) were adopted when outcome units were consistent across studies; standardized mean differences (SMD) were applied for outcomes with inconsistent units. The detailed unit information for each outcome is presented in Supplementary Table 3. For heterogeneity assessment, we employed the Q test and I^2^ statistic. A fixed-effects model was used when *P* ≥ 0.05 and I² < 50%, whereas a random-effects model was used when *P* < 0.05 and I² ≥ 50% (26). For this meta-analysis, *α*=0.05 was chosen as the significance level. Sensitivity analyses were carried out to examine the consistency of results for outcome markers with considerable heterogeneity (*P* < 0.05 or I^2^ ≥ 50%). To evaluate publication bias, we used funnel plots and Egger's regression test. We also used the trim-and-fill method to explore the potential influence of publication bias or small-study effects on the pooled estimates.

## 3 Results

The preliminary database search revealed 1,846 possibly eligible studies. After eliminating duplicate records and doing title/abstract screening, 277 research articles advanced to full-text eligibility evaluation. In total, 23 RCTs fulfilled all inclusion criteria and were incorporated into this meta-analysis: Zhang L (2023), Zhang L (2025), Wan SC (2024), Song WJ (2023), Chen TQ (2020), Hu Y (2024), Zhu SR (2025), Ye LZ (2022), Zhang YR (2023), Cao XH (2020), Liang Y (2022), Wang X (2021), Tao Y (2021), Zhao CY (2014), Ren W (2015), Li Q (2017), von Scholten BJ (2017), Zha M (2018), Huang MM (2021), Chen L (2025), Shi HT (2026), Guo RQ (2026) and Liu HM (2023) (27–49). Study selection procedures are detailed in Figure 1.

**Figure 1:**
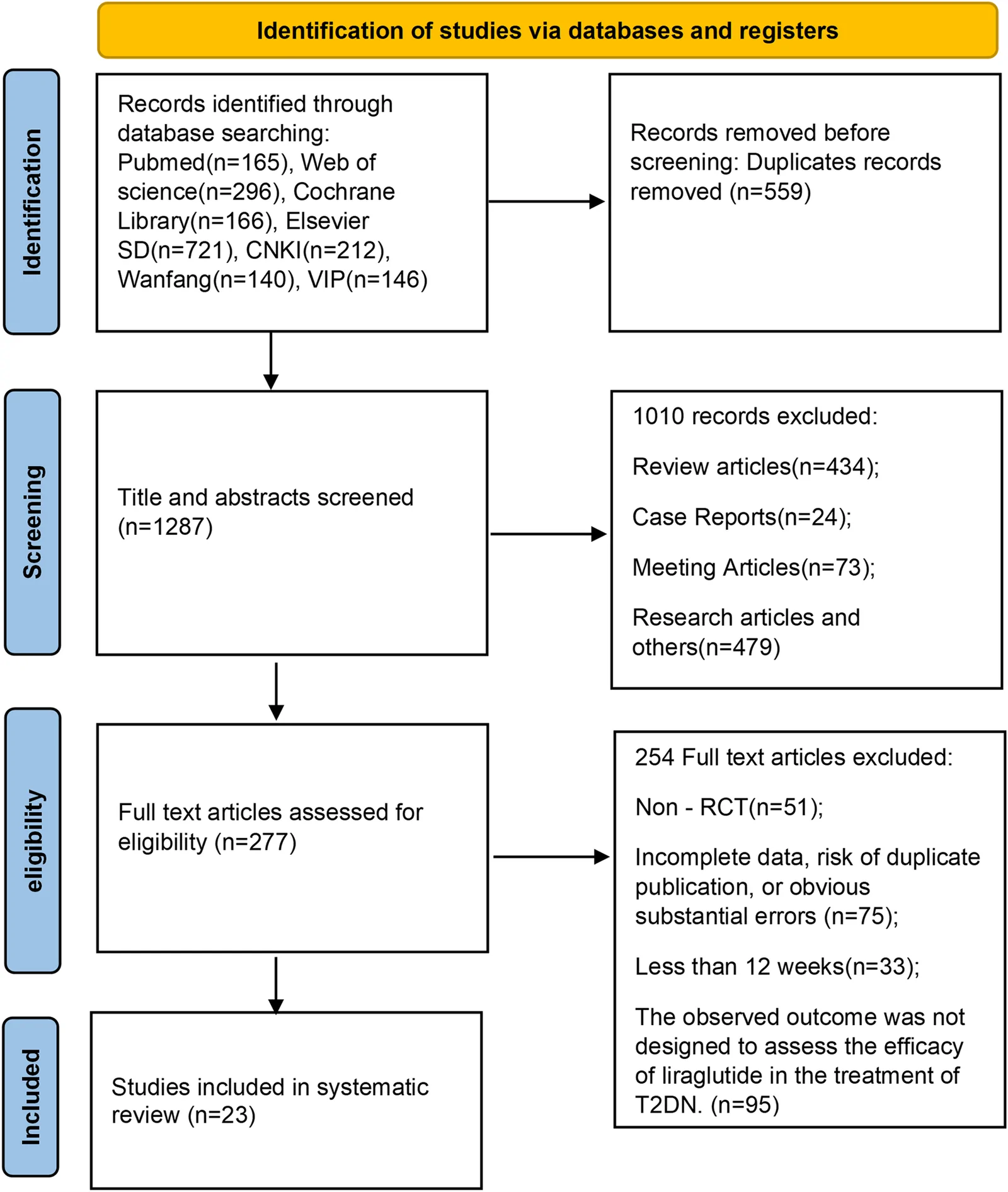
Flow plot.

### 3.1 Study characteristics

A total of 1,723 participants were included in 23 trials, with treatment durations ranging from 12 to 24 weeks. Interventions in the control group included conventional therapy, oral antidiabetic and antihypertensive medications, and insulin therapy. Participants in the intervention group received liraglutide alone or in combination with the same conventional therapy as that administered to the control group. Three trials had three arms, one had four arms, one used a randomized crossover design, and the remaining 18 were two-arm parallel-group trials. Detailed study characteristics are provided in Table 1 and Supplementary Tables 4, 5.

**Table 1:** Baseline data table: 1. LIR liraglutide, RT routine treatment, INS insulin; 2. A.SBP; B.DBP; C.HbA1c; D.UACR; E.BUN; F.Scr; G.eGFR; H.TC; I.TG; J.LDL-C; K.BMI; L.hs-CRP; M.Cys-C; N.*β*2-MG; 3. T, treatment group; C, control group.

Study author, year	Gender（male）T/C	Intervention measures		Sample sizeT/C	Therapy duration	Course of T2DM/year		Age T/C	Outcome Measures
		T	C			T	C		
Zhang L,2023 (27)	13/14	LIR(0.6 to 1.8 mg qd sc)	Metformin Tablets 0.5 g bid po, q7d increment 0.5 g, gradually titrate to 2 g/d po	30/30	12weeks	8.20 ± 4.32	8.30 ± 4.61	56.40 ± 5.35/57.93 ± 6.29	CDEFGK
Zhang L, 2025 (28)	17/16	LIR(1.8 mg qd sc) plus RT	RT	30/30	12weeks	8.65 ± 3.24	8.23 ± 3.12	61.25 ± 6.87/60.68 ± 7.02	ABCFGH
Wan SC, 2024 (29)	23/25	LIR(0.6 to 1.8 mg qd sc) plus INS	INS	43/43	12weeks	-	-	62.45 ± 8.96/63.11 ± 9.08	CDFHIJL
Song WJ, 2023 (30)	23/25	LIR(0.6 to 1.2 mg qd sc) plus Metformin Tablets	Metformin Tablets	80/80	12weeks	8.35 ± 4.36	8.25 ± 4.73	26.68 ± 3.12/26.62 ± 2.97	ABCDGHIJKLN
Chen TQ, 2020 (31)	39/40	LIR(0.6 to 1.8 mg qd sc) plus INS	INS	64/64	12weeks	7.40 ± 1.59	7.38 ± 1.57	55.20 ± 8.70/55.18 ± 8.67	CEFLM
Hu Y, 2024 (32)	29/30	LIR(0.6 to 1.8 mg qd sc) plus Sitagliptin	Sitagliptin Tablets 100 mg qd po	55/55	12weeks	5.32 ± 2.45	5.17 ± 2.36	55.22 ± 6.45/55.34 ± 6.52	CM
Zhu SR, 2025 (33)	17/18	LIR(0.6 to 1.2 mg qd sc) plus Metformin	Metformin Tablets 0.5-0.85 g bid po	30/30	12weeks	8.16 ± 1.40	8.51 ± 1.35	57.22 ± 4.09/56.45 ± 4.21	CDEFIJK
Ye LZ, 2022 (34)	26/28	LIR(0.6 to 1.8 mg qd sc) plus Dapagliflozin plus RT	Dapagliflozin Tablets 10 mg qd po plus RT	48/48	16weeks	7.02 ± 1.51	6.81 ± 1.29	52.93 ± 3.46/54.21 ± 4.78	ABCDEFGHIJK
Zhang YR, 2023 (35)	20/18	LIR(0.6 to 1.8 mg qd sc)	INS	30/30	12weeks	7.32 ± 0.32	7.35 ± 0.35	54.97 ± 10.42/54.82 ± 10.76	CMN
Cao XH, 2020 (36)	16/15	LIR(0.6 to 1.2 mg qd sc) plus Shenyan Kangfu Pian plus RT	Shenyan Kangfu Pian 5 tablets tid po plus RT	30/30	12weeks	4.08 ± 1.74	4.19 ± 1.52	55.74 ± 6.28/56.13 ± 6.91	CEFL
Liang Y, 2022 (37)	23/22	LIR(0.6 to 1.8 mg qd sc) plus Metformin plus Losartan Potassium	Metformin 0.5 g per dose bid po plus Losartan Potassium 50 mg per dose qd po	40/40	12weeks	4.96 ± 1.03	5.02 ± 1.09	72.68 ± 12.36/71.75 ± 12.24	CEF
Wang X,2021 (38)	16/16	LIR(0.6 to 1.8 mg qd sc) plus Metformin Hydrochloride Tablets/Acarbose Tablets	INS plus Metformin Hydrochloride Tablets/Acarbose Tablets	31/30	24weeks	8.43 ± 3.08	7.93 ± 2.23	56.10 ± 7.23/56.67 ± 6.13	CDGKN
Tao Y, 2021 (39)	28/24	LIR(0.6 to 1.8 mg qd sc) plus RT	INS plus RT	43/43	24weeks	9.74 ± 2.55	9.50 ± 2.81	67.4 ± 6.1/65.9 ± 6.9	CDFGHIJKM
Liu HM, 2023 (40)	22/24	LIR(0.6 mg qd sc)plus Gliquidone plus Irbesartan	Gliquidone Tablets 30 mg qd po plus Irbesartan Tablets 300 mg qd po plus Pioglitazone Tablets 15 mg qd po	40/40	12weeks	4.88 ± 1.14	4.35 ± 1.30	59.38 ± 4.39/60.72 ± 7.18	CEFHIJN
Zhao CY, 2014 (41)	-	LIR(0.6 to 1.8 mg qd sc) plus valsartan 80 mg qd po	INS+valsartan 80 mg qd po	19/26	24weeks	-	-	-	CKM
Ren W, 2015 (42)	-	LIR(0.6 to 1.8 mg qd sc) plus Losartan Potassium 50 mg qd po	INS plus Losartan Potassium 50 mg qd po	24/24	24weeks	-	-	-	CDFK
Li Q, 2017 (43)	11/13	LIR plus metformin 2 g/d	INS plus metformin 2 g/d	21/21	12weeks	4.20 ± 1.10	7.38 ± 1.50	48.20 ± 9.00/49.10 ± 8.30	ABCEFGHIK
von Scholten BJ, 2017 (44)	-	LIR(0.6 to 1.8 mg qd sc) plus standard therapy	placebo plus standard therapy	-	12weeks	-	-	-	ABCG
Zha M, 2018 (45)	-	LIR(0.6 to 1.8 mg qd sc) plus Huangkui Capsules 2.5 g tid	INS+Huangkui Capsules 2.5 g tid	30/30	24weeks	-	-	-	CDK
Huang MM, 2021 (46)	17/16	LIR(0.6 to 1.8 mg qd sc) plus INS	INS	32/32	12weeks	1.80 ± 0.30	1.70 ± 0.40	61.60 ± 1.20/61.50 ± 1.30	CEF
Chen L, 2025 (47)	27/26	LIR 0.6 mg qd sc plus dapagliflozin 10 mg qd po	Dapagliflozin 10 mg qd po	45/45	12weeks	0.82 ± 0.19	0.85 ± 0.17	51.76 ± 6.24/52.15 ± 6.64	EFN
Shi HT, 2026 (48)	17/16	LIR(0.6 to 1.8 mg qd sc) + metformin sustained-release 1 g/d po	Metformin sustained-release 1 g/d po	30/30	12weeks	-	-	63.35 ± 4.77/62.45 ± 4.65	CEFHIL
Guo RQ, 2026 (49)	27/25	LIR(0.6 to 1.2 mg qd sc) plus RT	RT	50/50	12weeks	10.50 ± 3.90	9.90 ± 4.40	57.80 ± 10.10/59.50 ± 9.20	ACDK

### 3.2 Risk of bias assessment

A comprehensive methodological quality assessment of the included research was conducted using the Cochrane Risk-of-Bias Tool. Among the 23 RCTs included, 12 adopted the random number table method, 1 employed the two-ball randomization method, and 1 utilized the random number ranking method. These studies were classified as low risk for random sequence development. Nine studies were rated as unknown risk for this category because they failed to clarify their randomization procedure. None of the included studies explicitly discussed allocation concealment procedures, and this domain was therefore assigned an unclear risk rating. One study (von Scholten BJ, 2017) reported the use of blinding and was rated as low risk, while the remaining studies were rated as unclear risk for the blinding domain. All studies provided complete and transparent outcome data, corresponding to a low risk rating for outcome data completeness. Additionally, all trials reported all pre-specified outcome measures without selective reporting, and no evidence of attrition bias was identified; these domains were also rated as low risk. Two studies (Wang X, 2021 and Ren W, 2015) were rated as high risk under the “Other Bias” domain, as they exclusively enrolled overweight or obese patients with T2DN. Given the weight-lowering and metabolic effects of liraglutide, this population restriction may have enriched participants who were more likely to respond favorably, potentially overestimating the treatment effect in the broader T2DN population. Detailed risk-of-bias profiles are presented in Figure 2 and Supplementary Figure 1.

**Figure 2:**
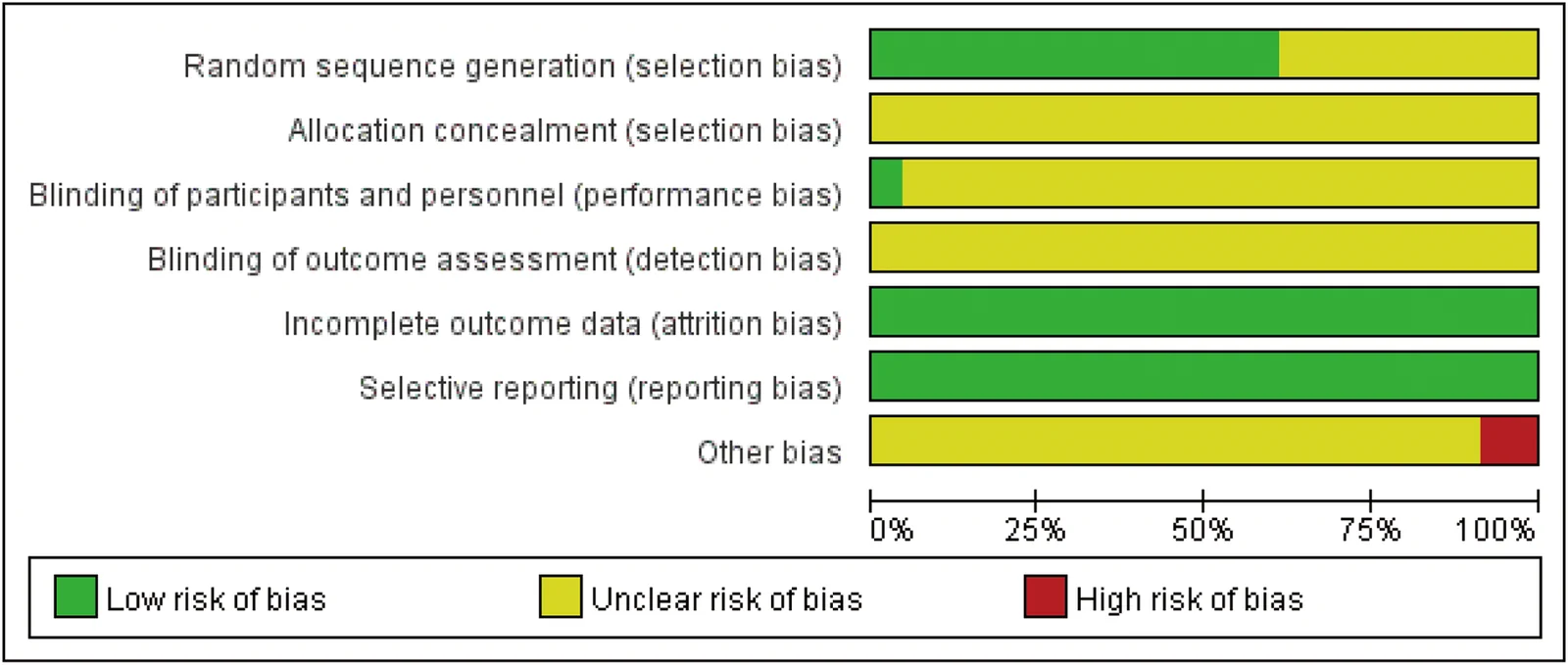
Risk of bias graph.

### 3.3 BP parameters

Five studies reported SBP, and four studies reported DBP. Meta-analytic findings demonstrated that, versus the control group, liraglutide significantly reduced patients’ SBP. The pooled effect estimate was as follows: SBP: MD = −5.00, 95% CI [−7.17, −2.84], I^2^ = 40%, *P* < 0.00001. No statistically significant between-group difference in DBP was observed (*P* = 0.15). The corresponding forest plots are presented in Figure 3.

**Figure 3:**
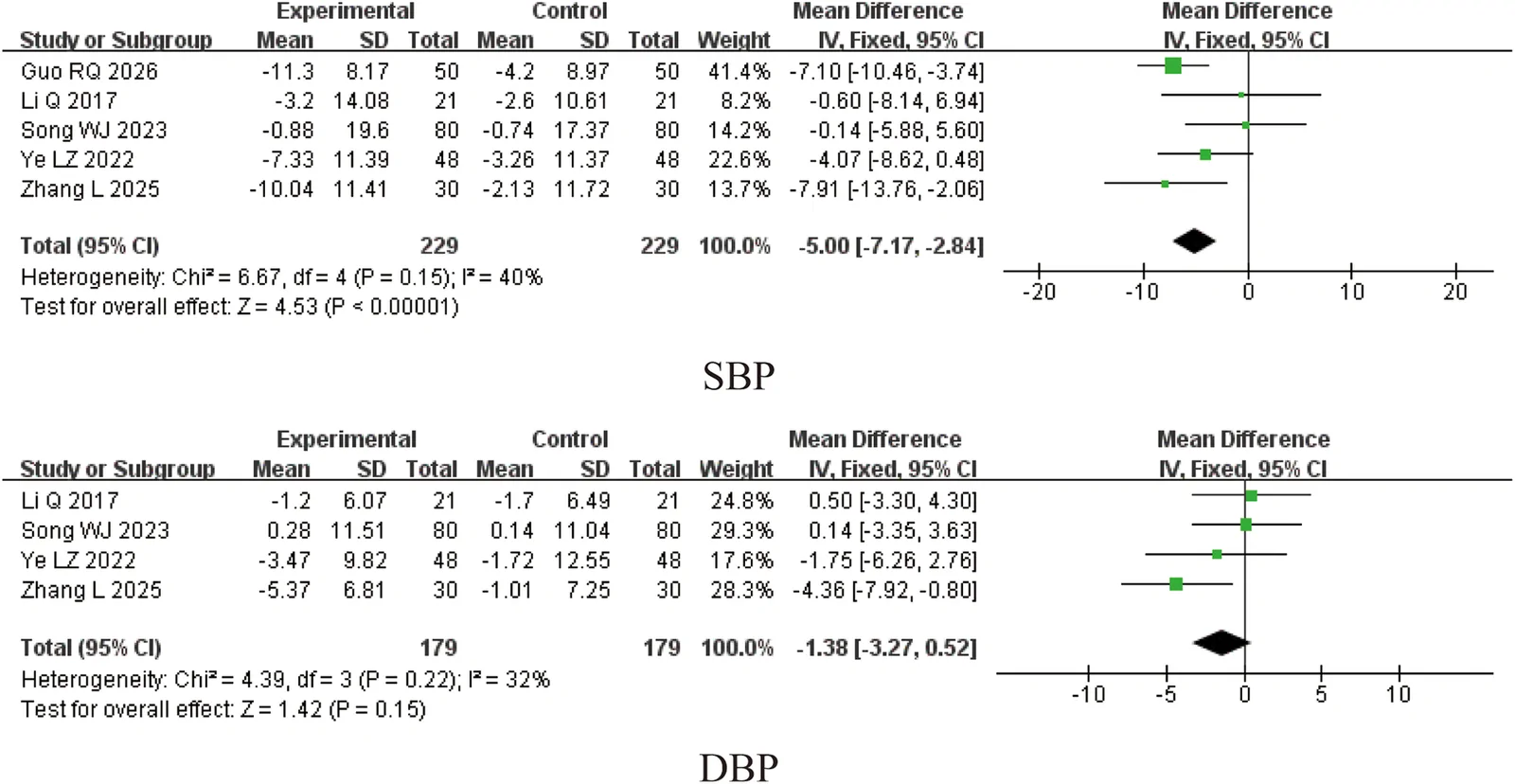
Forest plot of blood pressure parameters.

### 3.4 Renal function parameters

Seven studies reported eGFR, eleven reported BUN, fifteen reported Scr, five reported *β*2-MG, five reported Cys-C and ten reported UACR. Meta-analytic findings demonstrated that, versus the control group, liraglutide significantly preserved renal function in T2DN patients: specifically, it reduced UACR, Scr, Cys-C, BUN, and *β*2-MG levels and increased eGFR levels. The following were the corresponding pooled effect estimates: UACR: MD = −31.03, 95% CI [−41.47, −20.59], I^2^ = 94%, *P* < 0.00001; Scr: MD = −13.02, 95% CI [−20.63, −5.42], I^2^ = 96%, *P* = 0.0008; Cys-C: MD = −0.54, 95% CI [−0.84, −0.24], I^2^ = 87%, *P* = 0.0003; BUN: MD = −1.11, 95% CI [−1.75, −0.48], I^2^ = 91%, *P* = 0.0006; *β*2-MG: MD = −0.69, 95% CI [−1.33, −0.06], I^2^ = 91%, *P* = 0.03; eGFR: MD = 3.48, 95% CI [0.22, 6.73], I^2^ = 77%, *P* = 0.04. The corresponding forest plots are presented in Figure 4.

**Figure 4:**
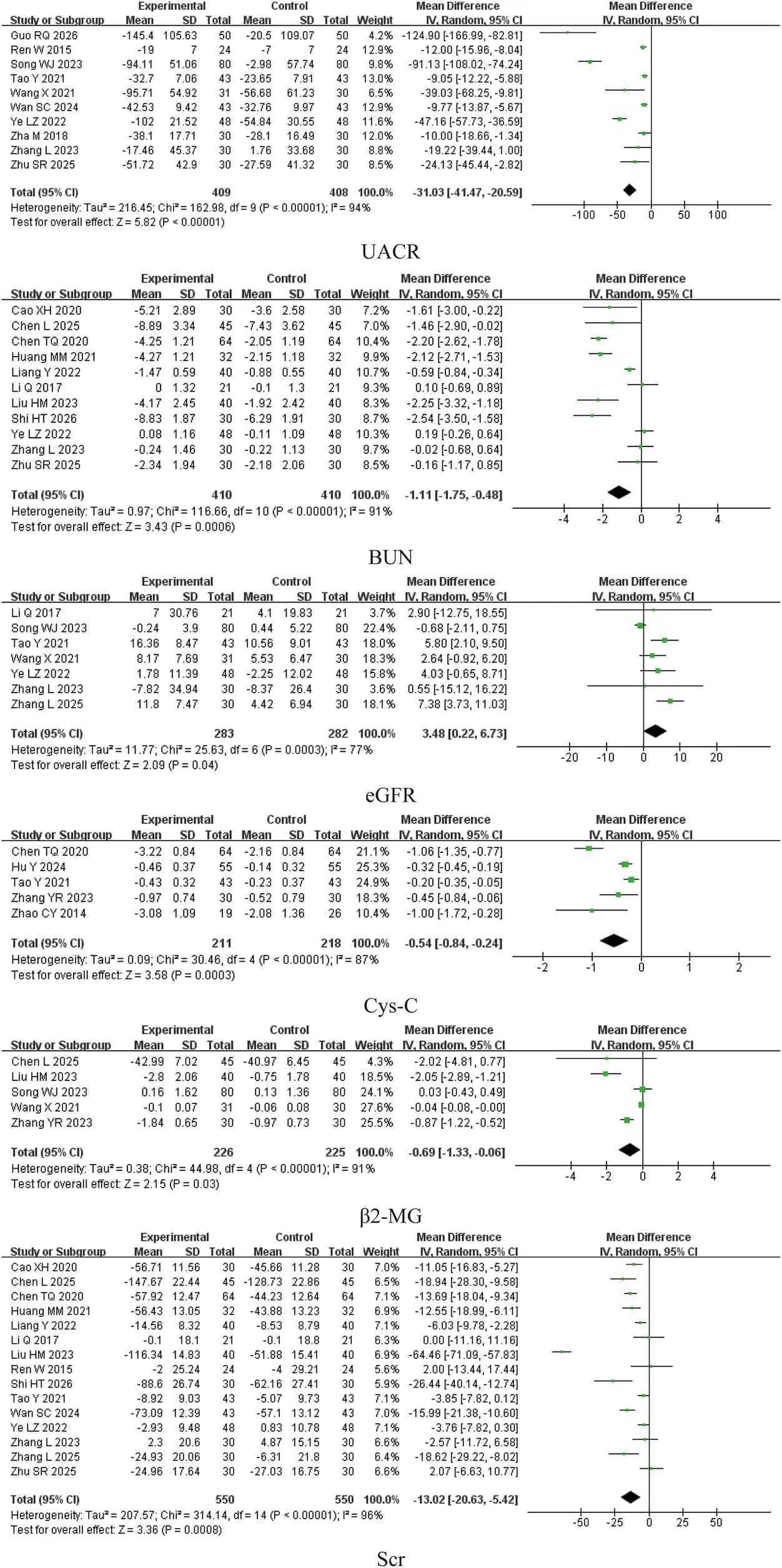
Forest plot of renal function parameters.

### 3.5 Blood glucose parameters

Twenty-one studies reported HbA1c. Meta-analytic findings revealed that, compared with the control group, liraglutide significantly reduced patients’ HbA1c. The pooled effect estimates were as follows: HbA1c: MD = −0.53, 95% CI [−0.80, −0.25], I^2^ = 87%, *P* = 0.0002. The corresponding forest plots are presented in Figure 5.

**Figure 5:**
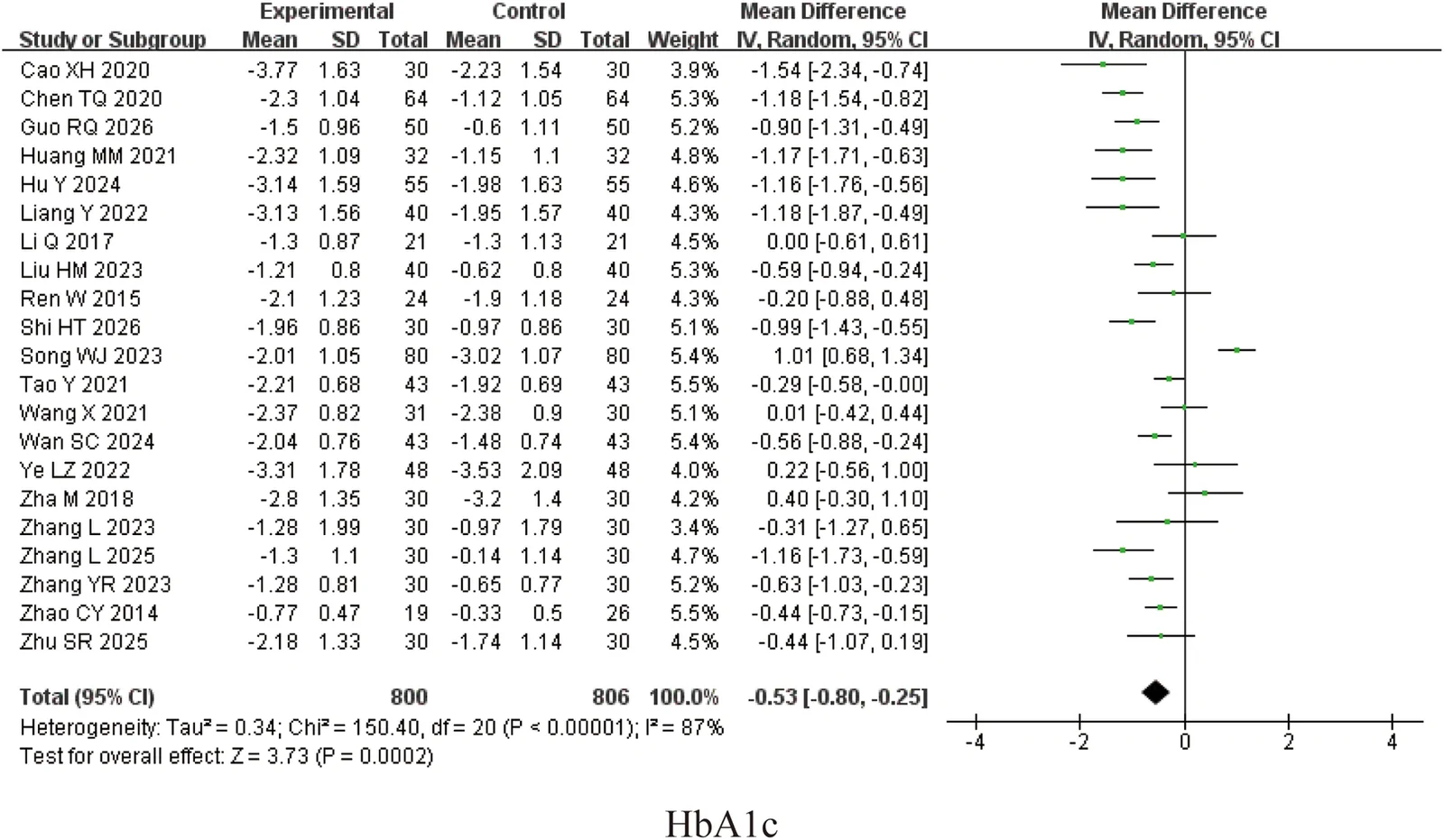
Forest plot of blood glucose parameters.

### 3.6 BMI and lipid metabolism parameters

Eight studies reported TC/TG, six LDL-C, and eleven BMI. Meta-analytic findings confirmed that liraglutide significantly lowered these indices in T2DN patients compared with the control group. The pooled effect estimates were as follows: TC: MD = −0.52, 95% CI [−0.85, −0.19], I^2^ = 78%, *P* = 0.002; TG: MD = −0.30, 95% CI [−0.47, −0.14], I^2^ = 74%, *P* = 0.0004; LDL-C: MD = −0.44, 95% CI [−0.62, −0.25], I^2^ = 58%, *P* < 0.00001; BMI: MD = −1.69, 95% CI [−2.39, −0.99], I^2^ = 90%, *P* < 0.00001. The corresponding forest plots are presented in Figure 6.

**Figure 6:**
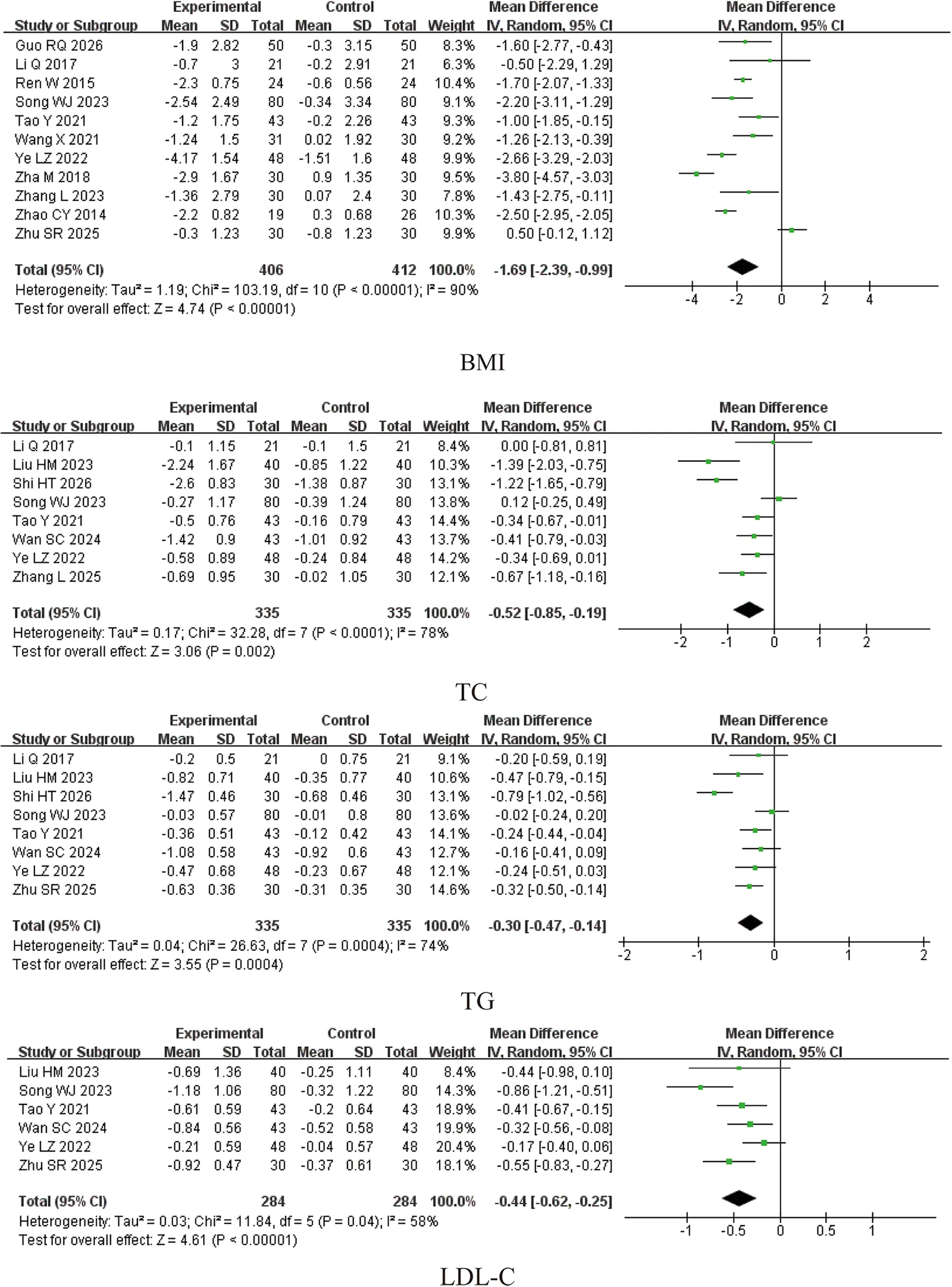
Forest plot of BMI and lipid metabolism parameters.

### 3.7 Inflammatory markers

Five studies reported hs-CRP. Meta-analytic findings demonstrated that, compared with the control group, liraglutide significantly reduced patients’ hs-CRP. The pooled effect estimates were as follows: hs-CRP: MD = −3.15, 95% CI [−5.08, −1.23], I^2^ = 96%, *P* = 0.001. The corresponding forest plots are presented in Figure 7.

**Figure 7:**
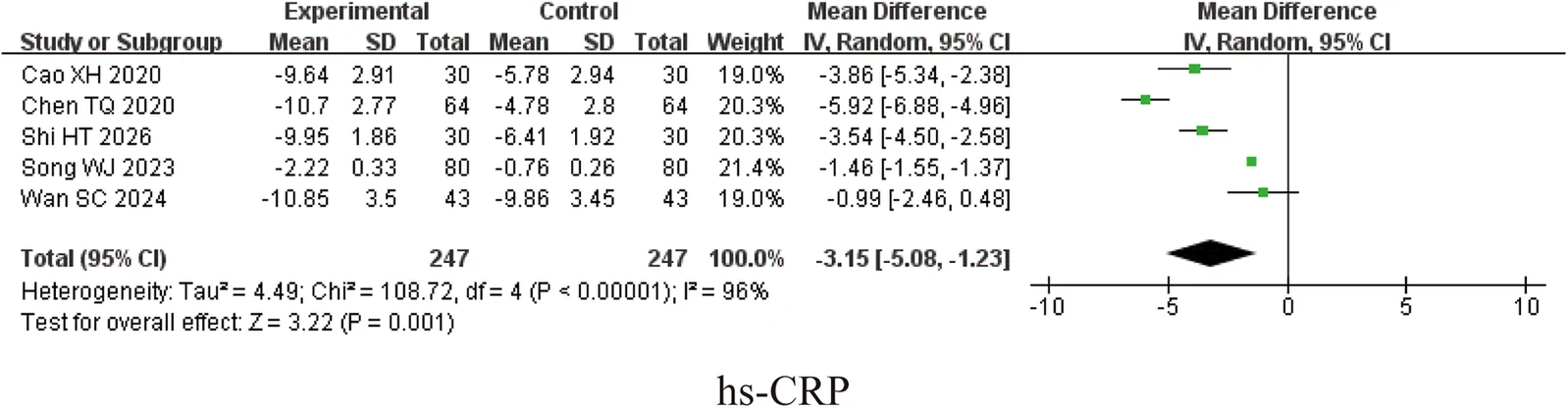
Forest plot of inflammatory markers.

### 3.8 Sensitivity analysis and publication bias

For all pooled outcomes, a leave-one-out strategy was used for sensitivity analysis. The results showed that, after excluding one study (Chen TQ, 2020), the heterogeneity for Cys-C was reduced (I^2^ decreased from 87% to 51%), without affecting the statistical significance of the results. The pooled effect size for Cys-C remained stable [MD = −0.32, 95% CI (−0.49, −0.16); *P* = 0.0001], indicating that Chen TQ (2020) was possibly a major source of heterogeneity. In addition, for the Scr outcome, heterogeneity decreased after excluding Liu HM (2023) (I^2^ decreased from 96% to 76%), and the pooled effect size remained stable [MD = −9.10, 95% CI (−12.62, −5.57); *P* < 0.00001]. For the remaining outcomes, exclusion of individual studies did not materially change the direction or statistical significance of the pooled estimates, although substantial heterogeneity persisted in several analyses.

To further explore potential sources of heterogeneity, subgroup analyses were performed according to the maintenance dose of liraglutide, intervention regimen, and baseline albuminuria category. For the dose-based analysis, studies were classified as low-dose (≤1.2 mg/d) or high-dose (>1.2 mg/d) according to the reported maintenance dose of liraglutide. For the intervention-regimen analysis, studies were classified as active-comparator therapy or add-on therapy. For the baseline albuminuria analysis, studies were classified as A2 (moderately increased albuminuria) or A3 (severely increased albuminuria). Subgroup analyses were performed only when at least two studies were available in each subgroup. In the analyses stratified by liraglutide maintenance dose, significant between-subgroup differences were observed for UACR and LDL-C (*P* < 0.05), whereas no significant subgroup differences were detected for the other outcomes. In the analyses stratified by intervention regimen, there were significant subgroup differences for UACR and Scr (*P* < 0.05). Meanwhile, substantial reductions in heterogeneity were observed for HbA1c (I^2^ = 37%) and UACR (I^2^ = 30%) within the active-comparator therapy subgroup. In the analyses stratified by baseline albuminuria category, a significant between-subgroup difference was observed only for HbA1c (*P* < 0.05), whereas no significant subgroup differences were found for BMI or UACR (*P* > 0.05). See Supplementary Table 6 for detailed results.

Funnel plot results showed no obvious imbalance (see Figure 8). Evidence of publication bias for UACR was identified using Egger's regression test (*P* = 0.017), whereas the other outcomes showed no indication of publication bias (*P* > 0.05). For detailed information, see Figure 9. The trim-and-fill method was applied to assess the potential influence of this bias on the pooled effect size of UACR. The adjusted pooled effect size was MD = −37.947 [95% CI (−55.397, −20.497); *P* < 0.001], which further supported the robustness of the current study's conclusions. The corrected results identified one hypothetically missing study. See Supplementary Figure 2 for detailed results.

**Figure 8:**
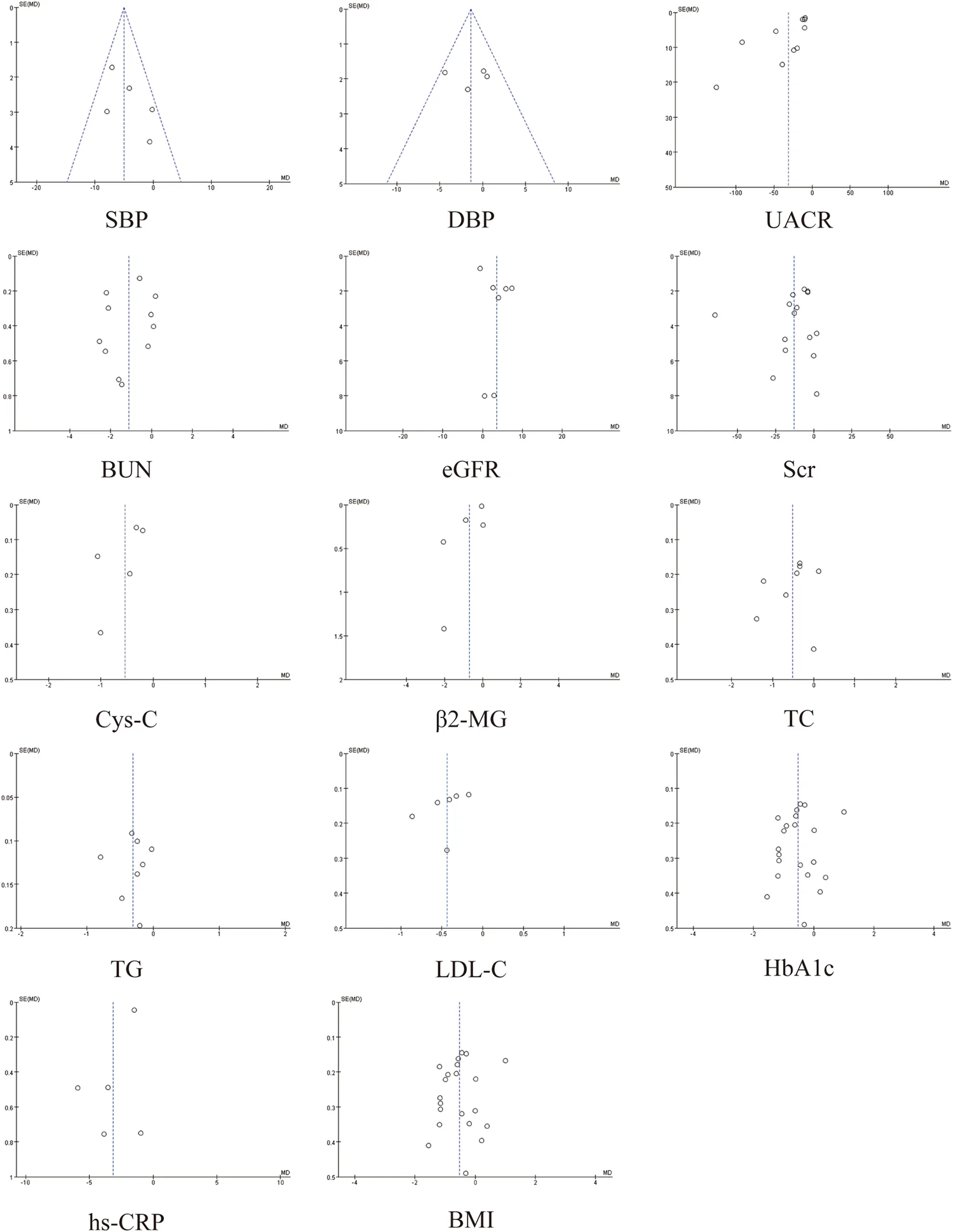
Funnel plots.

**Figure 9:**
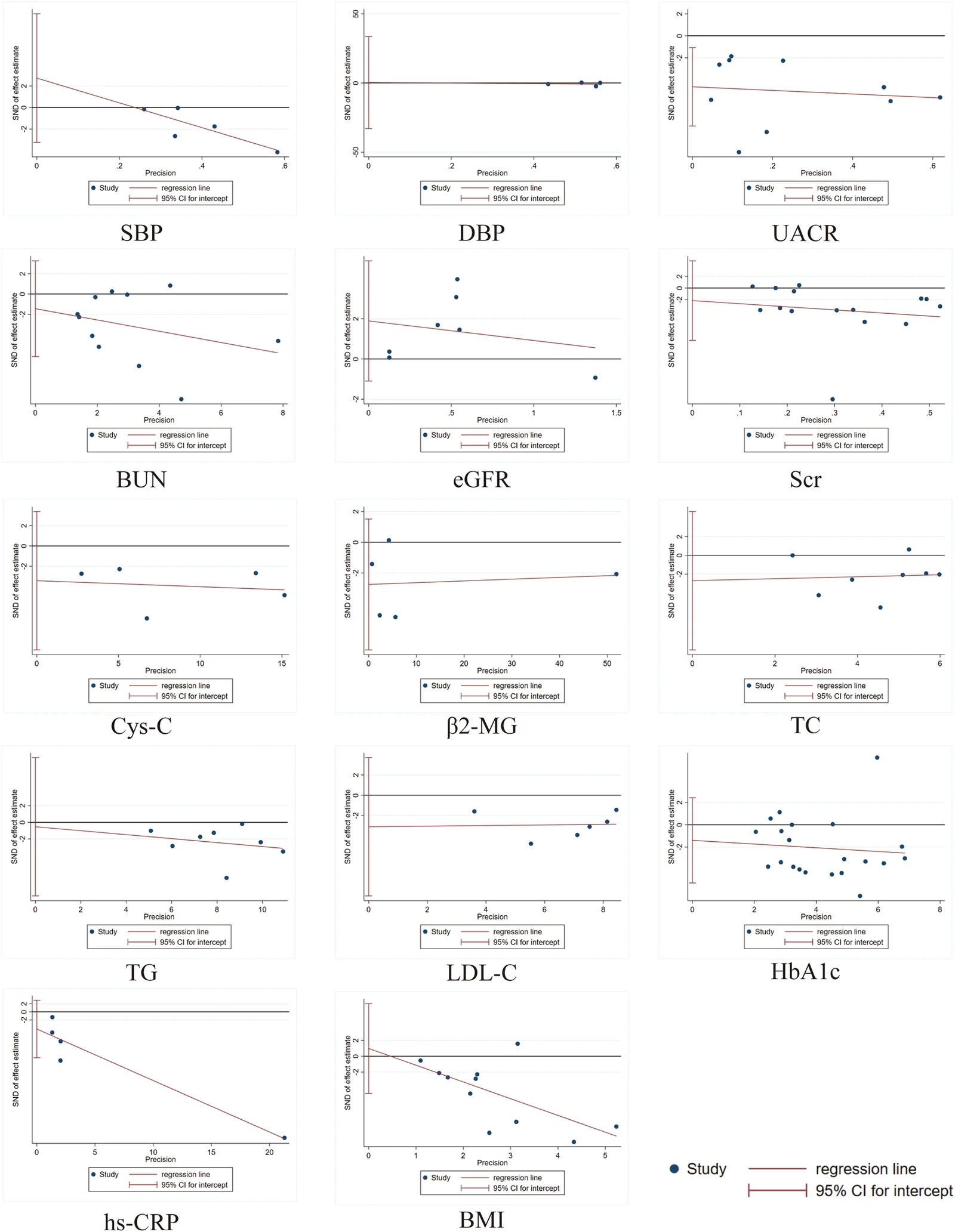
Egger plots.

## 4 Discussion

DN represents a major contributor to declining renal function and impaired quality of life among individuals with DM. Although novel therapeutic agents continue to emerge, no treatment has yet been shown to halt or reverse the progression of DN (50). In parallel with increasing obesity rates and population aging, the burden of diabetes and DN has risen markedly, and DN is now recognized as one of the principal causes of ESRD (51). DN is widely regarded as a chronic progressive microvascular disorder characterized by a range of metabolic and endocrine disturbances, including abnormalities in glucose metabolism, lipid profiles, and inflammatory responses (52). These interrelated abnormalities not only reflect disease progression but also further exacerbate renal injury (53). Hypertension is no exception; it is a key factor in the onset and progression of DN. In the early phase of the disease, glomerular hyperfiltration occurs, characterized by increased intraglomerular pressure and elevated renal blood flow (54). Persistent hyperglycemia and elevated BP further aggravate intraglomerular hypertension, leading to structural changes such as thickening of the glomerular basement membrane, ultimately resulting in proteinuria and progressive renal impairment. Reduction of BP helps alleviate intraglomerular pressure and exerts beneficial effects on the control of proteinuria and disease progression (55). Currently, ACEIs and ARBs are the most commonly used agents for controlling BP and proteinuria. However, there remains a need to explore additional therapies that can favorably influence BP, thereby enabling more individualized treatment strategies.

The primary benefit of liraglutide as a long-acting GLP-1RA is its pleiotropic, synergistic regulatory characteristics. Accumulating evidence suggests that liraglutide not only improves glycemic control but also contributes to weight reduction and renal protection (56, 57). Preclinical and clinical research have shown that liraglutide decreases the generation of inflammatory markers, modulates mesangial cell proliferation and activation, and downregulates the expression of inflammatory pathways like nuclear factor-kappa B (20). For lipid metabolic regulation, liraglutide suppresses the activity of sterol regulatory element-binding protein 1 and fatty acid synthase, important mediators of *de novo* lipid synthesis, while upregulating adipose triglyceride lipase and hormone-sensitive lipase, which are critical for the breakdown of fat (58). These two processes reduce ectopic lipid buildup in renal tubular epithelial cells. Importantly, liraglutide has been reported to exert hemodynamic effects. Liraglutide may lower BP partly by enhancing natriuresis and inhibiting the RAAS (59); however, its specific antihypertensive effects in patients with T2DN remain to be fully elucidated.

This study represents the first meta-analysis assessing the effects of liraglutide on BP in T2DN. Given the chronic and progressive nature of diabetic nephropathy, studies with a treatment duration of less than 12 weeks were excluded. A minimum follow-up period of 12 weeks was considered necessary to allow adequate exposure to liraglutide and consistent assessment of renal and metabolic outcomes across trials. This study included 23 RCTs published from database inception to September 7, 2026, and evaluated not only BP and renal function but also inflammation, lipid profiles, and glycemic control. The results demonstrated that liraglutide could reduce SBP in T2DN, whereas DBP remained unchanged. This difference may be related to the underlying mechanisms of liraglutide. Specifically, liraglutide can reduce blood volume by inhibiting proximal tubular sodium reabsorption and promoting natriuresis (60). In addition, it may enhance vascular function and decrease arterial stiffness (61). However, both reductions in blood volume and decreases in arterial stiffness are likely to have a greater impact on SBP than on DBP. Regarding renal function, liraglutide demonstrated beneficial effects on UACR, Scr, *β*2-MG, BUN, eGFR and Cys-C. Furthermore, liraglutide exerted beneficial effects on glycemic control, inflammatory responses, and lipid profiles. In the crossover trial by von Scholten (2017), liraglutide reduced HbA1c by 8 mmol/mol. A 5-mmHg reduction in 24-hour SBP was observed when comparing within-period changes between treatments. Urinary albumin excretion was also reduced. Because of the paired crossover design, these data were summarized narratively rather than included in the conventional parallel-group meta-analysis. These findings further support the clinical application of liraglutide in T2DN and offer guidance for the design of future high-quality clinical trials.

Given the relatively high heterogeneity observed in some outcomes, sensitivity analyses were performed, and subgroup analyses were conducted according to different maintenance doses of liraglutide, different intervention-regimen types, and different baseline albuminuria status. Sensitivity analyses suggested that Chen TQ (2020) may be a potential source of heterogeneity for Cys-C. This may be partly related to differences in background therapy and treatment contrast, particularly the concomitant use of insulin in this trial. Liu HM (2023) may be a potential source of heterogeneity for Scr. Liu HM (2023) reported substantially higher baseline Scr levels than most of the other included trials, suggesting more impaired baseline renal function. The greater potential for absolute reduction in Scr among these participants may partly explain the markedly larger treatment effect observed in this study and its contribution to between-study heterogeneity. Nevertheless, substantial residual heterogeneity remained after exclusion of this study (I^2^ = 76%), indicating that additional clinical and methodological differences across trials were also likely involved. Subgroup analyses further indicated that UACR and LDL-C were substantially influenced by the dose of liraglutide. In addition, both UACR and Scr were affected by intervention-regimen type, while HbA1c was affected by baseline albuminuria category. Notably, UACR and HbA1c, which exhibited substantial overall heterogeneity, showed markedly reduced heterogeneity in the active-comparator treatment subgroup: the I^2^ for HbA1c decreased from 87% to 37%, while that for UACR decreased from 94% to 30%. These findings suggest that differences in intervention regimens may partly account for the high overall heterogeneity observed for these two outcomes. However, because of the limited number of studies, this finding should be interpreted as hypothesis-generating rather than confirmatory. Given the very high heterogeneity observed for BMI (I^2^ = 90%), hs-CRP (I^2^ = 96%), and *β*2-MG (I^2^ = 91%), the clinical applicability of these findings may be limited, and the results for these outcomes should therefore be interpreted with caution. Finally, robustness was examined using Egger's test, funnel plots, and the trim-and-fill method.

Safety reporting varied considerably across the included RCTs, so the available data were summarized descriptively. Liraglutide showed a generally acceptable safety profile, with gastrointestinal symptoms and hypoglycemia being the most frequently reported adverse events. Overall adverse-event rates were broadly similar between groups, although some studies noted more gastrointestinal events with liraglutide. Treatment discontinuation due to gastrointestinal intolerance was uncommon, while serious adverse events were rarely reported and were usually judged unrelated to treatment. These findings remain preliminary because of the limited number of studies and short follow-up durations, and larger studies with longer follow-up are needed to better characterize the safety of liraglutide.

This study has several limitations. First, the diagnostic criteria for T2DN were not consistently defined across the included studies. In some trials, detailed diagnostic definitions or disease staging were not clearly reported. Given that T2DN represents a spectrum of disease severity ranging from microalbuminuria to advanced renal impairment, such inconsistency may lead to clinical heterogeneity and reduce comparability across pooled findings. Second, inconsistencies in measurement units across studies required data standardization prior to pooling. Although appropriate unit conversions were applied, potential measurement variability cannot be entirely excluded. Third, most included studies were conducted in China and were predominantly single-center trials with small to moderate sample sizes; only one crossover trial was conducted outside China. This geographic concentration may limit the generalizability of the findings and may contribute to regional selection bias. Fourth, safety data were incompletely and inconsistently reported across studies, particularly for treatment discontinuations and serious adverse events, precluding quantitative synthesis and firm conclusions regarding safety. Finally, the strength of evidence for several key outcomes remains limited. For example, only four studies reported DBP, thereby limiting the statistical power and generalizability of the pooled effect estimates.

In light of these limitations, we propose several directions. First, large-scale, multicenter RCTs with longer follow-up are needed to determine whether the observed short- to medium-term effects of liraglutide are sustained over time. Second, to improve the comparability of study data, greater emphasis should be placed on standardized study design and methodological transparency. This includes the use of uniform diagnostic criteria and clearly defined staging of T2DN, as well as comprehensive reporting of baseline patient characteristics, blinding procedures, and disease stages. Finally, future studies should focus on enhancing mechanistic investigations of liraglutide to clarify the principal signaling mechanisms that facilitate its diverse benefits in T2DN.

## 5 Conclusions

In the treatment of T2DN, liraglutide was associated with a modest reduction in SBP, while no statistically significant difference was observed for DBP. Liraglutide was also associated with favorable changes in selected renal biomarkers, lipid profiles, glycemic indices, BMI, and inflammatory markers. These findings should be interpreted cautiously because the DBP analyses were based on only four studies, and several outcomes were affected by substantial heterogeneity. Further well-designed randomized controlled trials are needed to clarify the effects of liraglutide on BP and renal outcomes in T2DN.

## Data Availability

The original contributions presented in the study are included in the article/Supplementary Material, further inquiries can be directed to the corresponding author.
