# Supplementary material for: Effects of liraglutide on blood pressure and renal function in patients with type 2 diabetic nephropathy: a meta-analysis of randomized controlled trials
Source: Front Med (Lausanne). 2026 Oct 9;13:1921737. doi: 10.3389/fmed.2026.1921737 (PMC13690535; doi:10.3389/fmed.2026.1921737)
Supplement: Supplementary file 1 [file Table1.docx]

Supplementary Table 1.Search strategy

PubMed：

Search: ((((((((liraglutide) OR (NN2211)) OR (Victoza)) OR (Saxenda)) OR ((((liraglutide[MeSH Terms]) OR (NN2211[MeSH Terms])) OR (Victoza[MeSH Terms])) OR (Saxenda[MeSH Terms]))) AND ((((((((((Diabetic Nephropathies) OR (Diabetic Kidney Disease)) OR (Diabetic Kidney Diseases)) OR (Diabetic Nephropathy)) OR (Diabetic Glomerulosclerosis)) OR (Intracapillary Glomerulosclerosis)) OR (Kimmelstiel Wilson Disease)) OR (Nodular Glomerulosclerosis)) OR (Kimmelstiel Wilson Syndrome)) OR (((((((((Diabetic Nephropathies[MeSH Terms]) OR (Diabetic Kidney Disease[MeSH Terms])) OR (Diabetic Kidney Diseases[MeSH Terms])) OR (Diabetic Nephropathy[MeSH Terms])) OR (Diabetic Glomerulosclerosis[MeSH Terms])) OR (Intracapillary Glomerulosclerosis[MeSH Terms])) OR (Kimmelstiel Wilson Disease[MeSH Terms])) OR (Nodular Glomerulosclerosis[MeSH Terms])) OR (Kimmelstiel Wilson Syndrome[MeSH Terms])))) ) )

Web of Science

liraglutide OR NN2211 OR Victoza OR Saxenda (All Fields) AND Diabetic Nephropathies OR Diabetic Kidney Disease OR Diabetic Kidney Diseases OR Diabetic Nephropathy OR Diabetic Glomerulosclerosis OR Intracapillary Glomerulosclerosis OR Kimmelstiel Wilson Disease OR Nodular Glomerulosclerosis OR Glomerulosclerosis, Nodular OR Kimmelstiel Wilson Syndrome (All Fields)

Elsevier ScienceDirect

('liraglutide' OR 'nn2211' OR 'victoza' OR 'saxenda') AND ('diabetic nephropathies' OR 'diabetic kidney disease' OR 'diabetic kidney diseases' OR 'diabetic nephropathy' OR 'diabetic glomerulosclerosis' OR 'intracapillary glomerulosclerosis' OR 'kimmelstiel wilson disease' OR 'nodular glomerulosclerosis' OR 'glomerulosclerosis, nodular' OR 'kimmelstiel wilson syndrome')

Cochrane Library:

liraglutide OR NN2211 OR Victoza OR Saxenda (Title Abstract Keyword) AND Diabetic Nephropathies OR Diabetic Kidney Disease OR Diabetic Kidney Diseases OR Diabetic Nephropathy OR Diabetic Glomerulosclerosis OR Intracapillary Glomerulosclerosis OR Kimmelstiel Wilson Disease OR Nodular Glomerulosclerosis OR Glomerulosclerosis, Nodular OR Kimmelstiel Wilson Syndrome (Title Abstract Keyword)

CNKI

（主题：利拉鲁肽）AND（主题：糖尿病肾病）

VIP

(题名或关键词=利拉鲁肽 AND 题名或关键词=糖尿病肾病)

Wanfang

主题:(利拉鲁肽) and 主题:(糖尿病肾病) 文献类型：期刊论文

Searches were conducted from database inception to September 7, 2026.

Supplementary Table2.

Formulas for Estimating Standard Deviation (SD) in Studies

| For studies reporting only pre-treatment and post-treatment values | SD = √(SD1² + SD2² - 2×r×SD1×SD2), r defaults to 0.5 |
| --- | --- |

Supplementary Table 3. Unit conversions applied to continuous outcomes

| Study | Outcome | Original unit | Standardized unit | Conversion formula used | Conversion required | Scale equivalence | Effect measure |
| --- | --- | --- | --- | --- | --- | --- | --- |
| Zhang L, 2023 | HbA1c | % | % | None | No | Identical scale | MD |
|  | UACR | mg/g | mg/g | None | No | Identical scale | MD |
|  | BUN | mmol/L | mmol/L | None | No | Identical scale | MD |
|  | Scr | μmol/L | μmol/L | None | No | Identical scale | MD |
|  | eGFR | ml/min/1.73 m² | ml/min/1.73 m² | None | No | Identical scale | MD |
|  | BMI | kg/m^2^ | kg/m^2^ | None | No | Identical scale | MD |
| Zhang L, 2025 | SBP | mmHg | mmHg | None | No | Identical scale | MD |
|  | DBP | mmHg | mmHg | None | No | Identical scale | MD |
|  | HbA1c | % | % | None | No | Identical scale | MD |
|  | Scr | μmol/L | μmol/L | None | No | Identical scale | MD |
|  | eGFR | ml/min/1.73 m² | ml/min/1.73 m² | None | No | Identical scale | MD |
|  | TC | mmol/L | mmol/L | None | No | Identical scale | MD |
| Wan SC, 2024 | HbA1c | % | % | None | No | Identical scale | MD |
|  | UACR | mg/g | mg/g | None | No | Identical scale | MD |
|  | Scr | μmol/L | μmol/L | None | No | Identical scale | MD |
|  | TC | mmol/L | mmol/L | None | No | Identical scale | MD |
|  | TG | mmol/L | mmol/L | None | No | Identical scale | MD |
|  | LDL-C | mmol/L | mmol/L | None | No | Identical scale | MD |
|  | hs-CRP | mg/L | mg/L | None | No | Identical scale | MD |
| Song WJ, 2023 | HbA1c | % | % | None | No | Identical scale | MD |
|  | TG | mmol/L | mmol/L | None | No | Identical scale | MD |
|  | LDL-C | mmol/L | mmol/L | None | No | Identical scale | MD |
|  | TC | mmol/L | mmol/L | None | No | Identical scale | MD |
|  | SBP | mmHg | mmHg | None | No | Identical scale | MD |
|  | DBP | mmHg | mmHg | None | No | Identical scale | MD |
|  | BMI | kg/m² | kg/m² | None | No | Identical scale | MD |
|  | UACR | mg/g | mg/g | None | No | Identical scale | MD |
|  | eGFR | ml/min/1.73 m² | ml/min/1.73 m² | None | No | Identical scale | MD |
|  | hs-CRP | mg/L | mg/L | None | No | Identical scale | MD |
|  | β2-MG | mg/L | mg/L | None | No | Identical scale | MD |
| Chen TQ, 2020 | HbA1c | % | % | None | No | Identical scale | MD |
|  | BUN | mmol/L | mmol/L | None | No | Identical scale | MD |
|  | Scr | μmol/L | μmol/L | None | No | Identical scale | MD |
|  | hs-CRP | mg/L | mg/L | None | No | Identical scale | MD |
|  | Cys-C | mg/L | mg/L | None | No | Identical scale | MD |
| Hu Y, 2024 | HbA1c | % | % | None | No | Identical scale | MD |
|  | Cys-C | mg/L | mg/L | None | No | Identical scale | MD |
| Zhu SR, 2025 | HbA1c | % | % | None | No | Identical scale | MD |
|  | UACR | mg/g | mg/g | None | No | Identical scale | MD |
|  | BUN | mmol/L | mmol/L | None | No | Identical scale | MD |
|  | Scr | μmol/L | μmol/L | None | No | Identical scale | MD |
|  | TG | mmol/L | mmol/L | None | No | Identical scale | MD |
|  | LDL-C | mmol/L | mmol/L | None | No | Identical scale | MD |
|  | BMI | kg/m² | kg/m² | None | No | Identical scale | MD |
| Ye LZ, 2022 | SBP | mmHg | mmHg | None | No | Identical scale | MD |
|  | DBP | mmHg | mmHg | None | No | Identical scale | MD |
|  | HbA1c | % | % | None | No | Identical scale | MD |
|  | UACR | mg/g | mg/g | None | No | Identical scale | MD |
|  | BUN | mmol/L | mmol/L | None | No | Identical scale | MD |
|  | Scr | μmol/L | μmol/L | None | No | Identical scale | MD |
|  | eGFR | ml/min/1.73 m² | ml/min/1.73 m² | None | No | Identical scale | MD |
|  | TC | mmol/L | mmol/L | None | No | Identical scale | MD |
|  | TG | mmol/L | mmol/L | None | No | Identical scale | MD |
|  | LDL-C | mmol/L | mmol/L | None | No | Identical scale | MD |
|  | BMI | kg/m² | kg/m² | None | No | Identical scale | MD |
| Zhang YR, 2023 | HbA1c | % | % | None | No | Identical scale | MD |
|  | Cys-C | mg/L | mg/L | None | No | Identical scale | MD |
|  | β2-MG | mg/L | mg/L | None | No | Identical scale | MD |
| Cao XH, 2020 | HbA1c | % | % | None | No | Identical scale | MD |
|  | BUN | mmol/L | mmol/L | None | No | Identical scale | MD |
|  | Scr | μmol/L | μmol/L | None | No | Identical scale | MD |
|  | hs-CRP | mg/L | mg/L | None | No | Identical scale | MD |
| Liang Y, 2022 | HbA1c | % | % | None | No | Identical scale | MD |
|  | BUN | mmol/L | mmol/L | None | No | Identical scale | MD |
|  | Scr | μmol/L | μmol/L | None | No | Identical scale | MD |
| Wang X, 2021 | HbA1c | % | % | None | No | Identical scale | MD |
|  | UACR | mg/g | mg/g | None | No | Identical scale | MD |
|  | eGFR | ml/min/1.73 m² | ml/min/1.73 m² | None | No | Identical scale | MD |
|  | BMI | kg/m² | kg/m² | None | No | Identical scale | MD |
|  | β2-MG | mg/L | mg/L | None | No | Identical scale | MD |
| Tao Y, 2021 | HbA1c | % | % | None | No | Identical scale | MD |
|  | UACR | mg/g | mg/g | None | No | Identical scale | MD |
|  | Scr | μmol/L | μmol/L | None | No | Identical scale | MD |
|  | eGFR | ml/min/1.73 m² | ml/min/1.73 m² | None | No | Identical scale | MD |
|  | TC | mmol/L | mmol/L | None | No | Identical scale | MD |
|  | TG | mmol/L | mmol/L | None | No | Identical scale | MD |
|  | LDL-C | mmol/L | mmol/L | None | No | Identical scale | MD |
|  | BMI | kg/m² | kg/m² | None | No | Identical scale | MD |
|  | Cys-C | mg/L | mg/L | None | No | Identical scale | MD |
| Liu HM, 2023 | HbA1c | % | % | None | No | Identical scale | MD |
|  | BUN | mmol/L | mmol/L | None | No | Identical scale | MD |
|  | Scr | μmol/L | μmol/L | None | No | Identical scale | MD |
|  | TC | mmol/L | mmol/L | None | No | Identical scale | MD |
|  | TG | mmol/L | mmol/L | None | No | Identical scale | MD |
|  | LDL-C | mmol/L | mmol/L | None | No | Identical scale | MD |
|  | β2-MG | mg/L | mg/L | None | No | Identical scale | MD |
| Zhao CY, 2014 | HbA1c | % | % | None | No | Identical scale | MD |
|  | BMI | kg/m² | kg/m² | None | No | Identical scale | MD |
|  | Cys-C | mg/L | mg/L | None | No | Identical scale | MD |
| Ren W, 2015 | HbA1c | % | % | None | No | Identical scale | MD |
|  | Scr | μmol/L | μmol/L | None | No | Identical scale | MD |
|  | UACR | mg/g | mg/g | None | No | Identical scale | MD |
|  | BMI | kg/m² | kg/m² | None | No | Identical scale | MD |
| Li Q, 2017 | HbA1c | % | % | None | No | Identical scale | MD |
|  | TC | mmol/L | mmol/L | None | No | Identical scale | MD |
|  | TG | mmol/L | mmol/L | None | No | Identical scale | MD |
|  | BUN | mmol/L | mmol/L | None | No | Identical scale | MD |
|  | Scr | μmol/L | μmol/L | None | No | Identical scale | MD |
|  | eGFR | ml/min/1.73 m² | ml/min/1.73 m² | None | No | Identical scale | MD |
|  | BMI | kg/m² | kg/m² | None | No | Identical scale | MD |
|  | SBP | mmHg | mmHg | None | No | Identical scale | MD |
|  | DBP | mmHg | mmHg | None | No | Identical scale | MD |
| von Scholten BJ, 2017 | HbA1c | mmol/mol | % | Mean (%) = 0.09148 × mean (mmol/mol) + 2.152; SD (%) = 0.09148 × SD (mmol/mol) | Yes | Equivalent after IFCC→NGSP conversion | MD |
|  | SBP | mmHg | mmHg | None | No | Identical scale | MD |
|  | DBP | mmHg | mmHg | None | No | Identical scale | MD |
|  | TC | mmol/L | mmol/L | None | No | Identical scale | MD |
|  | TG | mmol/L | mmol/L | None | No | Identical scale | MD |
|  | BUN | mmol/L | mmol/L | None | No | Identical scale | MD |
|  | Scr | μmol/L | μmol/L | None | No | Identical scale | MD |
|  | eGFR | ml/min/1.73 m² | ml/min/1.73 m² | None | No | Identical scale | MD |
|  | BMI | kg/m² | kg/m² | None | No | Identical scale | MD |
| Zha M, 2018 | BMI | kg/m² | kg/m² | None | No | Identical scale | MD |
|  | HbA1c | % | % | None | No | Identical scale | MD |
|  | UACR | mg/g | mg/g | None | No | Identical scale | MD |
| Huang MM, 2021 | HbA1c | % | % | None | No | Identical scale | MD |
|  | Scr | μmol/L | μmol/L | None | No | Identical scale | MD |
|  | BUN | mmol/L | mmol/L | None | No | Identical scale | MD |
| Chen L, 2025 | Scr | μmol/L | μmol/L | None | No | Identical scale | MD |
|  | BUN | mmol/L | mmol/L | None | No | Identical scale | MD |
|  | β2-MG | mg/L | mg/L | None | No | Identical scale | MD |
| Shi HT, 2026 | HbA1c | % | % | None | No | Identical scale | MD |
|  | TC | mmol/L | mmol/L | None | No | Identical scale | MD |
|  | TG | mmol/L | mmol/L | None | No | Identical scale | MD |
|  | Scr | μmol/L | μmol/L | None | No | Identical scale | MD |
|  | BUN | mmol/L | mmol/L | None | No | Identical scale | MD |
|  | hs-CRP | mg/L | mg/L | None | No | Identical scale | MD |
| Guo RQ, 2026 | SBP | mmHg | mmHg | None | No | Identical scale | MD |
|  | HbA1c | % | % | None | No | Identical scale | MD |
|  | UACR | mg/g | mg/g | None | No | Identical scale | MD |
|  | BMI | kg/m² | kg/m² | None | No | Identical scale | MD |

Supplementary Table 4. Characteristics of included non-parallel two-group RCTs

| Study | Study design | Randomization | T2DN confirmed | Therapy duration | Clarification |
| --- | --- | --- | --- | --- | --- |
| Zhu SR, 2025 | Three-arm RCT | Random-number table | Yes | 3 months | Group B vs Group A used |
| Zhang YR, 2023 | Four-arm RCT | Random-number table | Yes | 12 weeks | Liraglutide arm vs insulin arm used |
| Zhang L, 2023 | Three-arm RCT | Random-number table | Yes | 12 weeks | Liraglutide arm vs conventional-treatment arm used |
| Cao XH, 2020 | Three-arm RCT | Random-number table | Yes | 12 weeks | Combined-treatment arm vs nephritis rehabilitation tablets arm used |
| von Scholten BJ, 2017 | Randomized, double-blind, placebo-controlled crossover RCT | Random allocation sequence (1:1) | Yes | 12 weeks/period; 4-week washout | Within-patient liraglutide vs placebo comparison used |

Supplementary Table 5. Baseline clinical and renal characteristics of included studies

| Study author, year | T2DN diagnostic criteria | Baseline UACR/UAER, T/C (A category) | Baseline eGFR, T/C, mL/min/1.73 m² (G category) | Disease stage |
| --- | --- | --- | --- | --- |
| Zhang L, 2023 | Chinese diagnostic criteria for early diabetic nephropathy; UACR 30–300 mg/g | UACR **75.29±51.98 / 74.69±27.36 mg/g (A2/A2)** | 128.48±37.20 / 115.96±25.91 (G1/G1) | Early stage |
| Zhang L, 2025 | T2DM with mild-to-moderate DN; eGFR 30–89 mL/min/1.73 m² | - | 45.32±6.55 / 45.32±6.55 (G3a/G3a) | Mild-to-moderate DN |
| Wan SC, 2024 | T2DM according to the 2019 Chinese primary-care guideline; DN according to the 2014 Expert Consensus | ACR **95.24±10.56 / 94.63±10.97 mg/g (A2/A2)** | - | Early stage |
| Song WJ, 2023 | 2020 Chinese T2DM guideline; CKD/DKD criteria; early DKD defined as CKD stages 1–2 and/or UACR 30–300 mg/g | UACR **148.65±58.42 / 150.67±56.96 mg/g (A2/A2)** | 96.31±4.08 / 95.84±5.67 (G1/G1) | Early stage |
| Chen TQ, 2020 | WHO criteria for T2DM and Mogensen criteria for DN | - | - | - |
| Hu Y, 2024 | 2020 Chinese T2DM guideline and Mogensen criteria for DN; UAER >200 μg/min or 24-h total urinary protein >0.5 g | UAER **234.94±28.42 / 240.81±30.87 μg/min (A3/A3 by baseline mean)** | - | Early stage (as reported) |
| Zhu SR, 2025 | T2DM; ≥2 ACR measurements >30 mg/g within 3–6 months and persistent eGFR <60 for 3 months | ACR **324.38±49.12 / 312.46±46.52 mg/g (A3/A3)** | - | Early stage (as reported) |
| Ye LZ, 2022 | WHO 1999 criteria for T2DM and 2019 Chinese DKD guideline; eGFR ≥45 and UACR ≥30 mg/g | UACR **185.27±22.61 / 178.60±28.36 mg/g (A2/A2)** | 82.40±12.33 / 83.79±10.76 (G2/G2) | - |
| Zhang YR, 2023 | Chinese Adult DKD Clinical Diagnosis Expert Consensus; early DKD defined as UACR 30–300 mg/g or UAER 30–300 mg/24 h | UAER **186.57±12.31 / 185.48±12.53 mg/24 h (A2/A2)** | - | Early stage |
| Cao XH, 2020 | 2014 Expert Consensus on Prevention and Treatment of DN | - | - | - |
| Liang Y, 2022 | 2021 Chinese Guideline for Prevention and Treatment of DKD | - | - | Early stage |
| Wang X,2021 | WHO 1999 criteria for T2DM; UACR ≥30 mg/g; eGFR <30 excluded | UACR **291.28±62.55 / 297.82±64.39 mg/g (A2/A2 by baseline mean)** | 72.57±8.22 / 71.38±6.99 (G2/G2) | - |
| Tao Y, 2021 | Chinese diagnostic criteria for T2DM and 2014 Expert Consensus on DN | ACR **67.52±8.14 / 65.06±8.85 mg/g (A2/A2)** | 77.84±8.14 / 79.20±8.55 (G2/G2) | Mogensen I–II |
| Liu HM, 2023 | Chinese T2DM guideline and Chinese Adult DKD Clinical Diagnostic Expert Consensus | - | - | - |
| Zhao CY, 2014 | WHO 1999 criteria for T2DM; UAER 30–300 mg/24 h or 20-200 μg/min | - | - | Early stage |
| Ren W, 2015 | WHO 1999 criteria for T2DM; UAER 30–300 mg/24 h or 20-200 μg/min | ACR 29±5 / 27±7 mg/g; A2 by eligibility criterion | - | Early stage |
| Li Q, 2017 | Early T2DN with 24-h mAlb 30–300 mg/24 h and eGFR ≥60 mL/min/1.73 m² | - | 94.3±25.9 / 87.7±18.7 (G1/G2) | Early stage |
| von Scholten BJ, 2017 | T2DM according to WHO criteria; persistent albuminuria (UACR ≥30 mg/g in ≥2 of 3 consecutive morning samples); eGFR ≥30 mL/min/1.73 m² | - | - | - |
| Zha M, 2018 | WHO 1999 criteria for T2DM; UAER 20-200 μg/min | UACR 94.5±19.5 / 95.2±17.4 mg/g (A2/A2); UAER 102.1±19.4 / 103.6±17.5 μg/min (A2/A2) | - | Early stage |
| Huang MM, 2021 | Specific diagnostic criteria were NR | - | - | - |
| Chen L, 2025 | Chinese Guideline for the Prevention and Treatment of Diabetic Kidney Disease (2021); other causes of renal injury were excluded | - | - | - |
| Shi HT, 2026 | WHO criteria for T2DN as reported; HbA1c >6.5% with laboratory evidence of microalbuminuria | 24-h UAER 205.36±35.71 / 206.42±35.64 mg/24 h (A2/A2) | - | Microalbuminuric DN |
| Guo RQ, 2026 | Current Chinese guideline criteria: history of T2DM with persistent increased urinary albumin excretion and/or reduced GFR; non-diabetic renal injury excluded | UACR 418.3±115.6 / 402.1±110.3 mg/g (A3/A3) | - | - |

Note: T, treatment group; C, control group. Albuminuria categories were defined as A1, A2, and A3 according to UACR thresholds of <30, 30–300, and >300 mg/g, respectively, or corresponding UAER thresholds of <30, 30–300, and >300 mg/24 h (<20, 20–200, and >200 μg/min). eGFR categories were defined as G1 ≥90, G2 60–89, G3a 45–59, G3b 30–44, G4 15–29, and G5 <15 mL/min/1.73 m². When a separate baseline table was unavailable, measurements obtained before initiation of the study intervention were treated as baseline values.

Supplementary Table 6.

Subgroup analyses stratified by different doses of liraglutide

Subgroup1: low-dose (≤1.2 mg/d) groups; Subgroup2: high-dose (>1.2 mg/d) groups

HbA1c

| Category | Subgroup1 | Subgroup2 | Between-group difference |
| --- | --- | --- | --- |
| liraglutide dosage | -0.46 [-1.36, 0.43] | -0.60 [-0.84, -0.36] | P=0.77 |

BMI

| Category | Subgroup1 | Subgroup2 | Between-group difference |
| --- | --- | --- | --- |
| liraglutide dosage | -1.07 [-2.92, 0.79] | -2.10 [-2.73, -1.46] | P=0.30 |

UACR

| Category | Subgroup1 | Subgroup2 | Between-group difference |
| --- | --- | --- | --- |
| liraglutide dosage | -78.06 [-132.56, -23.56] | -17.46 [-24.63, -10.29] | P=0.03 |

BUN

| Category | Subgroup1 | Subgroup2 | Between-group difference |
| --- | --- | --- | --- |
| liraglutide dosage | -1.34 [-2.34, -0.35] | -1.18 [-2.05, -0.32] | P=0.81 |

Scr

| Category | Subgroup1 | Subgroup2 | Between-group difference |
| --- | --- | --- | --- |
| liraglutide dosage | -9.28 [-19.92, 1.36] | -9.66 [-13.68, -5.64] | P=0.95 |

β2-MG

| Category | Subgroup1 | Subgroup2 | Between-group difference |
| --- | --- | --- | --- |
| liraglutide dosage | -1.19 [-2.94, 0.56] | -0.44 [-1.25, 0.38] | P=0.44 |

TC

| Category | Subgroup1 | Subgroup2 | Between-group difference |
| --- | --- | --- | --- |
| liraglutide dosage | -0.61 [-2.09, 0.87] | -0.58 [-0.89, -0.26] | P=0.96 |

TG

| Category | Subgroup1 | Subgroup2 | Between-group difference |
| --- | --- | --- | --- |
| liraglutide dosage | -0.25 [-0.50, -0.01] | -0.36 [-0.65, -0.07] | P=0.58 |

LDL-C

| Category | Subgroup1 | Subgroup2 | Between-group difference |
| --- | --- | --- | --- |
| liraglutide dosage | -0.64 [-0.84, -0.43] | -0.29 [-0.43, -0.15] | P=0.006 |

hs-CRP

| Category | Subgroup1 | Subgroup2 | Between-group difference |
| --- | --- | --- | --- |
| liraglutide dosage | -2.54 [-4.88, -0.20] | -3.53 [-6.09, -0.98] | P=0.57 |

SBP

| Category | Subgroup1 | Subgroup2 | Between-group difference |
| --- | --- | --- | --- |
| liraglutide dosage | -6.03 [-8.74, -3.33] | -5.52 [-9.11, -1.92] | P=0.82 |

Subgroup analyses stratified by intervention regimen

Subgroup1: Active-comparator therapy; Subgroup2: Add-on therapy

HbA1c

| Category | Subgroup1 | Subgroup2 | Between-group difference |
| --- | --- | --- | --- |
| Intervention regimen | -0.30 [-0.49, -0.12] | -0.74 [-1.22, -0.27] | P=0.09 |

BMI

| Category | Subgroup1 | Subgroup2 | Between-group difference |
| --- | --- | --- | --- |
| Intervention regimen | -1.86 [-2.57, -1.15] | -1.48 [-3.15, 0.19] | P=0.68 |

UACR

| Category | Subgroup1 | Subgroup2 | Between-group difference |
| --- | --- | --- | --- |
| Intervention regimen | -10.99 [-14.49, -7.49] | -56.18 [-90.06, -22.30] | P=0.009 |

eGFR

| Category | Subgroup1 | Subgroup2 | Between-group difference |
| --- | --- | --- | --- |
| Intervention regimen | 4.04 [1.54, 6.54] | 3.37 [-2.16, 8.90] | P=0.83 |

BUN

| Category | Subgroup1 | Subgroup2 | Between-group difference |
| --- | --- | --- | --- |
| Intervention regimen | -0.66 [-1.94, 0.61] | -1.29 [-2.05, -0.52] | P=0.41 |

Scr

| Category | Subgroup1 | Subgroup2 | Between-group difference |
| --- | --- | --- | --- |
| Intervention regimen | -3.04 [-6.42, 0.33] | -11.46 [-15.56, -7.35] | P=0.002 |

β2-MG

| Category | Subgroup1 | Subgroup2 | Between-group difference |
| --- | --- | --- | --- |
| Intervention regimen | -0.89 [-1.79, 0.00] | -0.52 [-2.29, 1.26] | P=0.71 |

TC

| Category | Subgroup1 | Subgroup2 | Between-group difference |
| --- | --- | --- | --- |
| Intervention regimen | -0.58 [-1.32, 0.15] | -0.49 [-0.92, -0.07] | P=0.83 |

TG

| Category | Subgroup1 | Subgroup2 | Between-group difference |
| --- | --- | --- | --- |
| Intervention regimen | -0.29 [-0.44, -0.13] | -0.31 [-0.56, -0.05] | P=0.89 |

LDL-C

| Category | Subgroup1 | Subgroup2 | Between-group difference |
| --- | --- | --- | --- |
| Intervention regimen | -0.42 [-0.65, -0.18] | -0.45 [-0.72, -0.18] | P=0.84 |

Subgroup analysis by baseline albuminuria category

Subgroup1: A2 (moderately increased albuminuria); Subgroup2: A3 (severely increased albuminuria)

HbA1c

| Category | Subgroup1 | Subgroup2 | Between-group difference |
| --- | --- | --- | --- |
| Baseline albuminuria category | -0.09 [-0.55, 0.37] | -0.85 [-1.21, -0.50] | P=0.01 |

BMI

| Category | Subgroup1 | Subgroup2 | Between-group difference |
| --- | --- | --- | --- |
| Baseline albuminuria category | -2.04 [-2.73, -1.35] | -0.49 [-2.54, 1.57] | P=0.16 |

UACR

| Category | Subgroup1 | Subgroup2 | Between-group difference |
| --- | --- | --- | --- |
| Baseline albuminuria category | -26.84 [-37.24, -16.44] | -72.81 [-171.51, 25.88] | P=0.36 |

supplementary Figure 1:

supplementary Figure 2:
